# Dental Education

**Published:** 1875-09

**Authors:** E. J. Waye


### DENTAL REGISTER.

VOL. XXIX.] SEPTEMBER, 1875. [NO. 9.

### DENTAL EDUCATION.

BY E. J. WAYE, D. D. S.

*Read before the Northern Ohio Dental Association upon retiring from the Presidency.*

This subject is one which to many men in the profession possesses but little interest, that is to say in a general sense; I believe that a very large majority favor education to an extent, for instance, most desire such an education as will best enable them to fill a tooth with skill and elegance, or to construct with nice mechanical ingenuity, possibly with artistic taste, an artificial denture. They may even go farther, and desire such knowledge as will assist them to diagnose the condition of a tooth, diseased, and also the best manner of treatment. But of that broader knowledge of all the parts, their connection and sympathy, their anatomy, physiology, pathology and chemistry, indeed all the wide domain of study embraced in a thorough dental education, for how few, comparatively, has it either interest or attraction. To illustrate; visit one of our dental societies. The subject for discussion, we will suppose to be operative dentistry; you will soon discover that there exists a lively interest in the subject, both speakers and listeners wide awake and anxious both to hear and be heard, and as the best methods of operating, the different materials in use, the various preparations of gold and its manipulations are in turn discussed, you will not fail to perceive an eager earnestness to hear and understand every new method. And so long as the purely practical operation of the office and laboratory are presented for their consideration, there will be found no lack of attention or interest, but on the contrary there will be invariably manifested, an earnest desire to know all that may be taught regarding the absorbing topic.

How is it when the subjects of chemistry, histology or microscopy, claim their attention? A very brief observation is sufficient to convince the most skeptical, that for them, these subjects possess but little interest. Nay! that they are not understood; and judging by the indifference manifested, are considered as of little practical importance, other, than as furnishing topics upon which grave and learned professors may discant, disagree and mystify their audience and each other, and that so far from being of any real value in the struggle for patronage and preferment in life, or of practical benefit in office is concerned all knowledge of them may very well be dispensed with.

Were this indifference and ignorance confined to those, whose only claim to dental knowledge was acquired in the office and laboratory, it would be less a matter of surprise; but such is not the fact. Graduates from dental colleges to an extent almost beyond credence, are to be found in this class; men who having had opportunities and facilities for the study of every department of dental science, have devoted nearly their entire attention to the acquirment of such as would soonest secure a living; contenting themselves with such a merely superficial smattering of the rest, as would enable them to pass an examination.

Now if this statement be true, (and that it is so, I appeal to every observing man,) is it not apparent, that there must be a defect in the system under which they are educated? Or if not in the system itself, may it not be due to the fact, that certain things which are believed to be the essentials, are studied, (I do not say taught,) to the neglect of others equally important, as constituting a part of a thorough dental education, though not perhaps to the attainment of immediate practice? A young man, a graduate who possesses more than ordinary skill in the practical details of the profession, said to me lately, "I had when in college, an exellent opportunity for the study of histology, but finding most of the students eager to acquire the operative and mechanical, they being considered the essentials, I devoted most of my time to this study, much to my regret, whenever I happen to be present where that subject is under discussion."

What is the inference? Not that Histology was not taught certainly, but that in some way not easily explained perhaps, it was not assigned that prominence which its importance in dental education would justify, if not demand, and also (does it not?) a lack of thoroughness in the closing examination?

The want of a complete education is by no means, the distinguishing characteristic of the dentist. The professions both of law and medicine are similarly afflicted, and are earnestly seeking for the remedy. A leading medical journal, remarking upon the declension of physicians in public esteem, states as one of the reasons therefore: "The yearly out pouring, from the colleges of semi educated men," and assigns as a cause of this "semi education." The evasion of regulations, using these words; "The *theorg* of medical education is, that the candidate studies a year and a half in the office of some practioner, and then attends two courses of lectures before he graduates, making in all, a term of three years. The *fact* is, that a large proportion of the candidates really study only one year and a half, and some only one year."

If this be true of medicine are there no grounds for suspecting a like evasion in dentistry?

The regulations which govern the requirments as to lecture and office pupilage, are similar, and the facilities for acquiring knowledge, and for evasion of regulations the same; why should we expect different results?

To the dental college more perhaps than to all other influences, do we as a profession owe whatever of scientifie knowledge and investigation, as well as professional standing we possess; Appreciating fully our indebtedness to this source, it is with greater regret, that anything whatever, tending to weaken or destroy an unquestioning confidence in these institutions, should be witnessed as the result of their teaching, or mentioned, save in the spirit of kindness and appreciation, and as seeking a remedy for existing defects, rather than as upbraiding for past short comings.

Whatever the reasons, the facts are that thoroughly educated dentists are the exception, and semi (or less) educated ones the rule. To the colleges, as the highest exponents of dental education do we first look, asking and expecting that, if there be defects, either in regulations or modes of teaching that they be promptly acknowledged an.l remedied, and having done that, it may not be amiss for us, as members of a profession, whose common aim and interest should be its elevation and advancement, to ask ourselves, how have our duties toward these institutions, through wh:ch mainly we expect its accomplishment, been performed? First, in regard to students.

The theory is, that three years pulpilage with some dentist of respectability, two courses of lectures, concluding with a satisfactory examination, constitutes a dental education, upon which the possessor may enter into practice, and aided by experience, become a dentist in the best sense of the term?

What is the practice?

Either for a consideration, or because an office boy is wanted, a young man is received as a student who neither, by natural gifts or education, is in any sense adapted to the profession.

His education consists in being office boy, bill collector, plate scourer, (including the more laborious and disagreeable duties of the laboratory,) and malleter.

Entering upon this pupillage, with little or no education, with no known aptitude, for the delicate and difficult manipulations, peculiar to dentistry, with no habits of thought or study, he remains the prescribed term, (which is longer or shorter as his usefulness or the consideration may determine,) and with certificate of pupillage duly signed, is handed over to the college in the confident expectation, that with such an office experience, and two full courses of lectures, he will come out perfected and finished, in the entire curriculum, as per college announcement.

In this connection, it must be remembered that neither in dental or medical colleges, are students submitted to any examination, no questions being asked as to previous education; and as an inevitable result, men are admitted and graduated, who are unable to speak or write the English language correctly, and who must labor under a very serious disadvantage in not being able to understand and comprehend much of the language either in the text book or the lecture.

With such material and such preparatory teaching as a foundation, is it fair or reasonable, to expect, that two courses of lectures, however thorough and exhaustive, will suffice to rear a professional superstructure, which shall honor the profession and bless the community?

The fact is that comparatively few dentists, in these days, are competent to the task of properly instructing students: They may, it is true, impart some knowledge of the manipulations, both operative and mechanical, but as to directing or assisting them in the various studies prescribed by the colleges,—how many themselves possess the knowledge adequate to its performance? And of those who do, what proportion will conscientiously and earnestly exert themselves to impart it?

Herein lies an evil which must be remedied, before we can rationally entertain high hopes for the coming dentist.

The college by offering a preparatory course of instruction in view of office pupillage, has taken an important step in the right direction; one which is believed to possess such advantages as will eventually result in its becoming universally adopted. Not the least important of which rests in the fact, that while the pecuniary interests of the preceptor might, and doubtless many times does, prompt him to employ the time of the student for his own emolument or convenience, rather than the others improvement,—in the college no such inducement exists; while there is the additional advantage of receiving such aid in prosecuting his studies, as is rarely attainable in a dental office.

All this is gain to the student. Let us see if the college is not benefited as well.

Instead of receiving its matriculants from the office of any and every dentist whose interest or inclination may prompt to take a student; it is enabled to select from the infirmary, young men, whose education, natural aptitude and application to study, eminently fit them to receive and profit by its instructions; and who when entered upon active practice, will honor the institution from whence they graduate, the profession to which they are admitted, and bless the community whose good fortune it may be to receive their skillful and benign ministrations.

In conclusion, it is believed that whenever the dental college shall be owned and controlled by the dentists of the state wherein located, and aided by a law which provides that only through its portals, may any man enter our noble profession, the conditions for the remedy of existing evils will have been secured; and that, in the future, the reproach of ignorance and incompetency, now, too frequently with justice, preferred against us as a profession, will cease to mortify our pride, weaken our influence and afflict community.

